# Increased skin conductance responses and neural activity during fear conditioning are associated with a repressive coping style

**DOI:** 10.3389/fnbeh.2015.00132

**Published:** 2015-06-02

**Authors:** Tim Klucken, Onno Kruse, Jan Schweckendiek, Rudolf Stark

**Affiliations:** ^1^Department of Psychotherapy and Systems Neuroscience, Justus Liebig University GiessenGiessen, Germany; ^2^Bender Institute of Neuroimaging, Justus Liebig University GiessenGiessen, Germany

**Keywords:** classical conditioning, fear, vigilance, sensitizer, amygdala, reappraisal

## Abstract

The investigation of individual differences in coping styles in response to fear conditioning is an important issue for a better understanding of the etiology and treatment of psychiatric disorders. It has been assumed that an avoidant (repressive) coping style is characterized by increased emotion regulation efforts in context of fear stimuli as compared to a more vigilant coping style. However, no study so far has investigated the neural correlates of fear conditioning of repressors and sensitizers. In the present fMRI study, 76 participants were classified as repressors or as sensitizers and were exposed to a fear conditioning paradigm, in which the CS+ predicted electrical stimulation, while another neutral stimulus (CS−) did not. In addition, skin conductance responses (SCRs) were measured continuously. As the main findings, we found increased neural activity in repressors as compared to sensitizers in the ventromedial prefrontal cortex and the anterior cingulate cortex (ACC) during fear conditioning. In addition, elevated activity to the CS+ in amygdala, insula, occipital, and orbitofrontal cortex (OFC) as well as elevated conditioned SCRs were found in repressors. The present results demonstrate increased neural activations in structures linked to emotion down-regulation mechanisms like the ventromedial prefrontal cortex, which may reflect the increased coping effort in repressors. At the same time, repressors showed increased activations in arousal and evaluation-associated structures like the amygdala, the occipital cortex (OCC), and the OFC, which was mirrored in increased SCRs. The present results support recent assumptions about a two-process model of repression postulating a fast vigilant response to fear stimuli, and a second process associated with the down-regulation of emotional responses.

## Introduction

Coping can be defined as the ability to process and deal with emotional stimuli, e.g. emotion regulation (Goldin et al., 2008). Dysfunctional coping processes in context of fear stimuli are associated with the etiology of psychiatric disorders (Goldin et al., 2014; Sheppes et al., 2015). Fear conditioning is an established model for the development, maintenance, and treatment of psychiatric disorders (Delgado et al., 2006; Schweckendiek et al., 2011). Thus, the identification of individual differences in coping styles, which impact fear conditioning, may lead to a better understanding of (dys)functional human behavior.

In fear conditioning paradigms, a neutral stimulus (CS+) is associated with an aversive stimulus (UCS) like electrical stimulation, while a second neutral stimulus (CS−) predicts the absence of the UCS. After a few pairings, increased conditioned responses (CRs) to the CS+ as compared to the CS− can be observed, e.g., elevated skin conductance responses (SCRs), startle response, changes in subjective ratings, and altered neural activity (Hamm and Weike, 2005; Dunsmoor et al., 2007; Klucken et al., 2009a, 2013a; Tabbert et al., 2011). Regarding the neural correlates of fear conditioning, many studies have identified a fear-network including the amygdala, the nucleus accumbens (NAcc), the anterior cingulate cortex (ACC), the insula, the orbitofrontal cortex (OFC), and the occipital cortex (OCC; Sehlmeyer et al., 2009; Klucken et al., 2012). Thereby, the amygdala plays an important role for the processing of CRs (LaBar et al., 1998). Beside the amygdala, recent studies suggest an involvement of the NAcc for the CS/UCS association (Klucken et al., 2009b; Pohlack et al., 2012; Do-Monte et al., 2013; Bulganin et al., 2014). In addition, blood oxygen level dependent (BOLD) signal change -responses in the (lateral) OFC, the ACC, and the insula have been considered as neural correlates of higher cognitive and interoceptive evaluation processes (O’Doherty, 2007; Caria et al., 2010; Lissek et al., 2014), while OCC activations are often associated with increased (motivated) attention (Bradley et al., 2003).

The coping model of Krohne and colleagues (Krohne et al., 2000) focuses on individual differences in coping styles when confronted with aversive stimuli and has been repeatedly associated with altered physiological (Rohrmann et al., 2003; Klucken et al., 2010), cognitive (Peters et al., 2012), and neural (Rauch et al., 2007, 2014; Paul et al., 2012) responses. In detail, two independent coping styles have gained increased attention: Subjects with a repressive coping style (“*repressors*”) are characterized by cognitive avoidance of fear stimuli to prevent the experience of arousal (Krohne et al., 2000). In contrast, “*sensitizers*” are supposed to exhibit vigilant, approaching behavior towards negative stimuli (Krohne et al., 2000). Notably, previous studies have reported a paradoxical dissociation effect by showing increased peripheral-physiological responses in repressors as compared to sensitizers, while sensitizers often reported increased subjective distress (Kohlmann et al., 1996; Derakshan and Eysenck, 2001; Rohrmann et al., 2002; Derakshan et al., 2007; for review see: Schwerdtfeger and Kohlmann, 2004; Rofé, 2008).

Regarding neural differences between repressors and sensitizers, current fMRI studies have shown increased responses in motivated-attention related areas like the OCC, but concurrently also in structures that are involved in the suppression and down-regulation of emotions, i.e., the (ventromedial) prefrontal cortex (vmPFC) and the ACC (Rauch et al., 2007, 2014; Paul et al., 2012; Raio and Phelps, 2015). Regarding the amygdala, the few existing results are inconsistent. Dysfunctional cognitive reappraisal was linked to increased amygdala volume in humans (Hermann et al., 2014a). Additionally, a trend-wise increased amygdala activation during the presentation of fearful faces in sensitizers (Rauch et al., 2007) or a negative correlation between amygdala activity and cognitive reappraisal (Hermann et al., 2014b) has been reported. However, another human study found no group differences in amygdala activity between repressors and sensitizers (Rauch et al., 2014). Regarding fear conditioning, only two studies have so far investigated group differences between repressors and sensitizers. The first study found increased conditioned SCRs in repressors as compared to sensitizers (Scarpetti, 1973), while the second study did not find group differences (Urban and Kohlmann, 1994). However, since the focus of the second study was extinction learning, only five CS/UCS pairings were used in the fear acquisition process.

The aim of the present study was to investigate group differences between repressors and sensitizers in fear conditioning as well as the underlying neural correlates. In accordance with the dissociation effect, we expected increased SCRs in repressors as compared to sensitizers. Based on the abovementioned findings, it was hypothesized that repressors would show higher vmPFC, ACC, OCC, and striatal activations in the contrast CS+ > CS− as compared to sensitizers, while amygdala differentiation was investigated exploratively.

## Materials and Methods

### Participants

For the present study, participants were classified as repressors (*n* = 37; 19 male; mean age: 24.7; SD: 4.91) or sensitizers (*n* = 42; 22 male; mean age: 23.1; SD: 2.76) using the Mainz Coping Inventory (MCI; Krohne et al., 2000). The MCI is a self-report questionnaire assessing different coping styles by asking for avoidance or vigilance strategies in different fear-relevant situations. In order to include only subjects with a clearly defined coping style, repressors were defined by percentile ranks above 50 on the gender-specific “cognitive avoidance” scale and below 50 on the gender-specific “vigilance” scale, while sensitizers were defined by percentile ranks above 50 on the “vigilance” and below 50 on the “cognitive avoidance” scale. Other coping styles were not included in the analysis due to the lack of clear hypotheses (Krohne et al., 2000). Current or past mental, sexual, or chronic health problems as well as consumption of psychotropic drugs were defined as exclusion criteria. All participants were right-handed, had normal or corrected-to-normal vision, and received 40 Euro for their participation. Participants gave an informed consent. The study was conducted in accordance with the Declaration of Helsinki and was approved by the institutional ethics committee. Three participants (two repressors) were excluded due to excessive (> 6 mm) head motion during scanning, leaving 76 participants in the final sample.

### Conditioning Procedure

A differential fear conditioning procedure (each CS: 16 trials) was conducted using colored squares as reinforced conditioned (CS+) or as non-reinforced (CS−) stimuli. Electrical stimulation was used as unconditioned stimulus (UCS; 50% reinforcement). Each CS was presented for 8 s. The UCS (duration= 100 ms) was delivered 7.9 s after the CS+ onset and co-terminated with the CS+ offset. The inter-trial-interval (ITI) ranged from 4.5 to 7 s. Electrodes were fixed to the middle of the left shin and stimulus intensity was set individually to an “unpleasant but not painful” sensation. A custom-made impulse-generator (833 Hz) provided transcutaneous electrical stimulation (UCS) for 100 ms through two Ag/AgCl electrodes (1 mm^2^ surface). Because the experiment is part of a larger project investigating fear conditioning, genetics, and different extinction procedures, two different colored CS+ were used. The two CS+ did neither differ significantly in valence, arousal, or UCS−expectancy ratings nor in SCRs (all *p* > 0.700) or hemodynamic responses, and are therefore analyzed together. Different extinction techniques were also assessed but will be reported elsewhere. The stimuli were projected onto a screen at the back of the scanner (visual field = 18°) using an LCD projector and were viewed through a mirror mounted to the head coil. An MRI-compatible video camera was used to check whether participants watched the stimuli.

### Skin Conductance Measures

SCRs were recorded during the complete MR scan using Ag/AgCl electrodes [filled with isotonic (0.05 M NaCl) electrolyte medium] placed hypothenar at the non-dominant (left) hand. The largest difference between a minimum value, which had to occur within a 1–8 s time window after the CS (CS+ or CS−) onset, and the following maximum was counted as the entire interval response (EIR; Pineles et al., 2009). Statistical analyses were performed via Analysis of variance (ANOVA) in a 2 (stimulus: CS+ vs. CS−) × 2 (group: repressors vs. sensitizers) × 2 (time: early phase vs. late phase) design followed by Bonferroni-corrected *post hoc* tests using SPSS 22 (IBM Corporation, Armonk, USA).

### Magnetic Resonance Imaging

#### Hemodynamic Activity

All images were acquired with a 1.5 Tesla whole-body tomograph (Siemens Symphony with a quantum gradient system) with a CP head coil. Structural image acquisition consisted of 160 T1-weighted sagittal images (MPRage, 1 mm slice thickness; TR = 1.9 s; TE = 4.16 ms; field of view 250 × 250 mm). For functional images, a total of 292 images were registered using a T2*-weighted gradient echo-planar imaging (EPI) sequence with 25 slices covering the whole brain (5 mm slice thickness 1 mm gap; descending slice procedure; TR = 2.5 s; TE = 55 ms; flip angle = 90; field of view 192 × 192 mm; matrix size = 64 × 64; voxel size = 3 × 3 × 5 mm). The orientation of the axial slices was paralleled to the OFC tissue-bone transition. Data were analyzed using Statistical Parametric Mapping (SPM8, Wellcome Department of Cognitive Neurology, London UK; 2008) implemented in MATLAB 7.5 (Mathworks Inc., Sherbourn, MA). Prior to all statistical analyses, data were preprocessed as described before. The experimental conditions were CS+, CS−, UCS+, and UCS− (time corresponding to the UCS after the CS−). In line with the analyses of SCRs, regressors were also split into a first half (CS+_1_/CS−_1_; UCS+_1_/UCS−_1_) and a second half (CS+_2_/CS−_2_; UCS+_2_/UCS−_2_) to investigate potential group differences between the early and the late phase of fear conditioning (Straube et al., 2007; Klucken et al., 2015). All regressors were convolved with the hemodynamic response function. The six movement parameters of the rigid body transformation obtained by the realignment procedure were entered as covariates in the model. The voxel-based time series was filtered with a high pass filter (time constant = 128 s).

On the group level, two sample *t-*tests were conducted to examine differences between repressors and sensitizers for the contrast CS+ > CS−. Following a worthwhile reviewer’s comment, we also correlated fMRI data (CS+ − CS−) with SCRs responses for repressors and sensitizers. Whole brain analyses were conducted with *p* < 0.05 (family-wise-error corrected (FWE)) and *k* > 10 voxels. Region of interest (ROI) analyses were performed using the small volume correction in SPM8 *p* < 0.05 (FWE-corrected; *k* > 5 voxels).

ACC, amygdala, insula, NACC, OCC masks were taken from the “Harvard-Oxford cortical and subcortical structural atlases” provided by the Harvard Center for Morphometric Analysis. The lateral OFC mask was created with MARINA (Walter et al., 2003). Hermann and colleagues kindly provided the vmPFC mask (Hermann et al., 2012).

## Results

### Skin Conductance Responses

**Figure 1:**
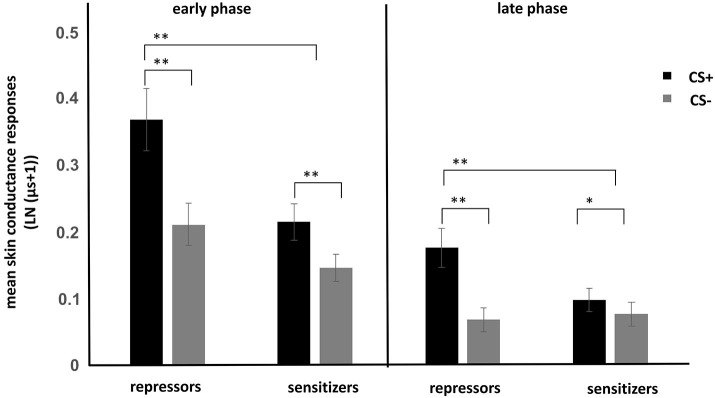
**Mean skin conductance responses to CS+ and CS− for repressors and sensitizers for the early (first half of conditioning) and late phase (second half of conditioning)**. Enhanced conditioned responses (CRs) were found in the repressor group as compared to the sensitizer group. *p < 0.05. **p < 0.01.

ANOVA showed a significant main effect of stimulus regarding the EIR (*F*_(1,74)_ = 75.84, *p* < 0.001) revealing increased SCRs to the CS+ as compared to the CS−. In addition, main effects of time (*F*_(1,74)_ = 86.23, *p* < 0.001) and group (*F*_(1,74)_ = 4.72, *p* < 0.05) were observed. More important, a significant stimulus × group interaction (*F*_(1,74)_ = 18.67, *p* < 0.001) was observed. *Post hoc* tests showed that conditioning was successful in both groups (Figure 1), which is reflected in increased SCRs to the CS+ as compared to the CS− (all *p* < 0.05). Repressors demonstrated stronger CRs than sensitizers in the early as well as in the late phase.

### fMRI-Results

#### Main Effect of Stimulus (CS+ > CS−)

Since the main aim of the study was to investigate differences between repressors and sensitizers, we will only briefly report the main effects of conditioning, which have been reported before Sehlmeyer et al. (2009). ROI-analyses revealed significant results in the contrast CS+ > CS− in all ROIs, further supporting successful CS+/CS− differentiation.

#### Group Differences in the Contrast CS+ > CS−

In accordance with the SCRs, a significant association of neural activations with coping style was observed. Whole brain results showed increased activations in the prefrontal cortex (*x/y/z* = 16/62/19, *z*_max_ = 5.05, *p* < 0.01) in repressors as compared to sensitizers for the complete conditioning phase. Regarding the early phase of fear conditioning (CS+1 − CS−1), increased activation was found in repressors as compared to sensitizers in the limbic lobe (middle temporal gyrus) (*x/y/z* = 36/11/−32, *z*_max_ = 4.86, *p* < 0.05). In addition, a trend was found in the later phase showing increased activations in repressors in the PFC (*x/y/z* = 18/62/19, *z*_max_ = 4.44, *p* < 0.1).

**Figure 2:**
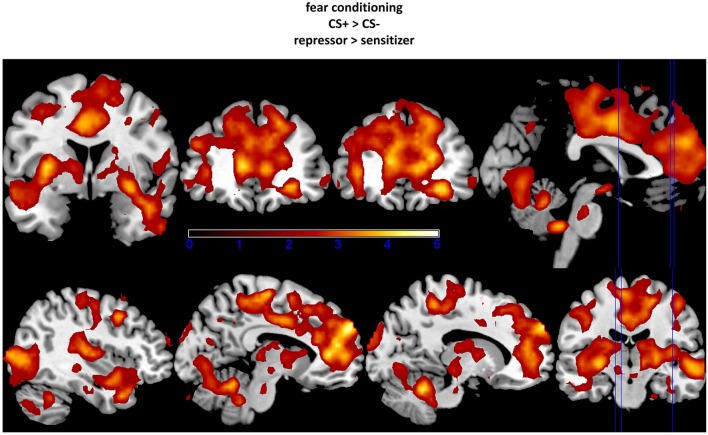
**Increased activation in amygdala, insula, and orbitofrontal cortex (OFC; upper part) as well as in occipital cortex (OCC), anterior cingulate cortex (ACC), and vmPFC (lower part) in repressors as compared to sensitizers (CS+ − CS−)**. The fixation point for each region was set at the peak voxel. Data were thresholded with a *t* = 2.0.

ROI-analyses further revealed stronger neural activations in the ACC, the insula, the lateral OFC, the OCC, and the vmPFC in repressors compared to sensitizers over the complete conditioning phase as well as increased amygdala activity in the early phase (see Table 1; Figure 2). In addition, no increased activations could be found in sensitizers as compared to repressors. Finally, we correlated SCR data with BOLD-responses for each group separately and found a (trendwise) link between amygdala and SCR activations (*x/y/z* = −18/−13/−14, *k* = 18 *z*_max_ = 2.74, *p* = 0.058).

**Table 1:** **ROI-activations (CS+ > CS−) for fear conditioning (whole phase)**.

Group analysis	Contrast	Structure	Side	*k*	*x*	*y*	*z*	*z*_max_	*p* corr
Repressors	CS+ >	ACC	L	650	−9	41	7	4.00	0.008
Sensitizers	CS−	Amygdala*	R	14	30	2	−14	2.63	0.072
		Insula	L	253	−33	2	7	3.86	0.006
		Insula	R	138	36	8	−14	3.67	0.012
		Lateral OFC	L	436	−27	23	−14	4.47	0.001
		OCC	R	275	39	−91	7	3.70	0.040
		vmPFC	L	111	−12	53	−2	3.30	0.044

Region of Interest Analyses, threshold = p < 0.05 (FWE-corrected; small volume correction according to SPM8). Results are displayed until p < 0.08. All coordinates refer to MNI space. L, left hemisphere; R, right hemisphere. *significant in the early phase of fear conditioning.

## Discussion

The present study demonstrated that repressors and sensitizers differ in CRs. In detail, repressors displayed higher conditioned SCRs during fear conditioning as compared to sensitizers. In addition, we also found increased neural activations in repressors as compared to sensitizers in the contrast CS+ − CS− in coping-relevant structures like the vmPFC and the ACC as well as in arousal- and emotion-relevant structures like the amygdala, the OCC, the insula, the NAcc, and the OFC. The data support the view that repressors are characterized by vigilance and down-regulation processes in response to fear stimuli.

Regarding vigilance processes, increased amygdala activity is often assumed as neural correlate for an increased sensitivity to the CS+, because amygdala activation constitutes an important process for the stabilization of the learning signal and initiating psychophysiological CRs (Delgado et al., 2006). In addition, insula and OFC activations have been linked to detailed stimulus processes during fear conditioning (Sehlmeyer et al., 2009). Altered occipital activation is often interpreted as a neural correlate of increased attention (Bradley et al., 2003). For instance, enhanced occipital activation has been found during acute presentation of visually aversive stimuli, but also occurs during their anticipation. This has often been referred to as increased motivated attention (Bradley et al., 2003; Ueda et al., 2003; Tabbert et al., 2010; Merz et al., 2012a; Klucken et al., 2013b). This supports the assumption that activation within the OCC is not only stimulus driven, but can also result from top-down processes modulating attention to a stimulus. The increased SCRs together with the neural findings in repressors further underline vigilance processes to the CS+. SCRs are often interpreted as automatic responses to salient cues reflecting increased attention processes to these stimuli. Further, the present data is in line with previous results showing increased SCRs in repressors during fear acquisition (Scarpetti, 1973).

Regarding cognitive avoidance strategies in repressors, we found increased activations in the vmPFC and the ACC. While ACC activation is a common finding in fear conditioning, increased vmPFC activation during fear conditioning is surprising because most studies show an involvement during fear extinction but not during fear conditioning. Many studies linked vmPFC responses to fear inhibition (e.g., during fear extinction) and/or to emotion regulation (Milad et al., 2007; Goldin et al., 2008; Hermann et al., 2009; Merz et al., 2012b; Klucken et al., 2013b; Lissek et al., 2014). In detail, enhanced vmPFC BOLD-responses have been previously reported in other studies using different stimuli and designs (Eippert et al., 2007; Rauch et al., 2007, 2014; Paul et al., 2012), showing an involvement in emotion down-regulation (Goldin et al., 2008; Hermann et al., 2009; Paul et al., 2012), through inhibiting amygdala activity. For instance, one study demonstrated a relation between increased PFC activity during fear conditioning and down-regulation strategies (e.g., the imagination of a safe situation) that are similar to the preferred coping-mechanisms of repressors (Delgado et al., 2008). The altered vmPFC activation might thus reflect an increased effort to cope and regulate emotions in repressors as compared to sensitizers. With respect to clinical findings, the present findings (increased SCRs, and BOLD-responses in the fear circuit and vmPFC activations) may mirror the increased negative health outcome in repressors as compared to non-repressors (see Derakshan et al., 2007, for overview). In the review, Derakshan et al. (2007) speculated that the increased reactivity to fear stimuli with the (potential) coping attempt may lead to the increased negative health status in repressors. For instance, longitudinal studies showed a negative correlation between a repressive coping style and treatment success in somatic diseases (Frasure-Smith et al., 2002).

In addition, we found a trendwise significant correlation between amygdala and SCRs in repressors. Previous studies showed that the amygdala is involved in the production of CRs (Petrovic et al., 2008). It is therefore assumable, that amygdala activation may reflect the outcome of fear conditioning and/or the strength of fear memory. However, the result was only a trend and not significant. Therefore, this argumentation should be treated with caution until an independent replication is available.

### Future Directions

In the present study, participants were not explicitly instructed to use emotion-regulation strategies. This is in line with previous studies with repressors and sensitizers (Rauch et al., 2007, 2014) investigating trait differences in coping styles, rather than state-induced emotion regulation techniques. It therefore has to be kept in mind that the described role of the vmPFC is a *post hoc* explanation of the observed results. Future studies could investigate if repressors cope more extensively during fear conditioning. In addition, it would be interesting to investigate whether the opposite pattern of results would emerge if sensitizers were instructed to use avoidance strategies, while repressors were instructed to use vigilance coping mechanisms. Finally, some studies showed that vigilance-avoidance responses in repressors are especially visible during very fast responses (e.g., the first 500 ms after stimulus onset) for every trial, and not over the whole experiment like we found (Derakshan et al., 2007). Using fMRI and BOLD-responses, we were not able to draw such conclusions. Finally, it should be noted that the present study used a fear conditioning design with two CS+. This procedure may lead to increased uncertainty, fear, and stress responses, which could lead to increased group differences and more effort for coping processing as compared to a differential fear conditioning design with one CS+ only. It is therefore interesting to investigate group differences using paradigms with different complexity and uncertainty to gain a better understanding of repression and sensitization.

In sum, the results clearly support the assumption that a person’s coping mode is associated with fear conditioning. We observed increased SCRs and BOLD-responses in subcortical and cortical structures in repressors as compared to sensitizers. These findings contribute to the current debate of the vigilance-avoidance model and provide potential neural mechanisms linked to vigilance and down-regulation processes in repressors.

## Conflict of Interest Statement

The authors declare that the research was conducted in the absence of any commercial or financial relationships that could be construed as a potential conflict of interest.
